# Phycocyanin Protects against High Glucose High Fat Diet Induced Diabetes in Mice and Participates in AKT and AMPK Signaling

**DOI:** 10.3390/foods11203183

**Published:** 2022-10-12

**Authors:** Shuai Hao, Fannian Li, Qiancheng Li, Qi Yang, Wenjing Zhang

**Affiliations:** Beijing Engineering and Technology Research Center of Food Additives, Beijing Technology and Business University, Beijing 100048, China

**Keywords:** phycocyanin, diabetes mellitus, SMMC-7721 cells, AKT and AMPK signaling, antidiabetic function

## Abstract

Phycocyanin is a type of marine natural product and functional food additive. Studies have demonstrated that phycocyanin has potential regulatory effects on glycometabolism, while its function and mechanism, especially in type 2 diabetes mellitus (T2DM), is still unclear. The aim of this study was to investigate the antidiabetic roles and underlying mechanism of phycocyanin in a high glucose high fat diet induced model of T2MD in C57BL/6N mice and a high-insulin-induced insulin-resistant model of SMMC-7721 cells. The results indicated that phycocyanin reduced high glucose high fat diet induced hyperglycemia as well as ameliorated glucose tolerance and histological changes in the liver and pancreas. Meanwhile, phycocyanin also significantly decreased the diabetes-induced abnormal serum biomarker variations, including triglyceride (TG), total cholesterol (TC), aspartate transaminase (AST), and glutamic-pyruvic transaminase (ALT), and increased the superoxide dismutase (SOD) content. Furthermore, the antidiabetic function of phycocyanin was exerted through activating the AKT and AMPK signaling pathway in the mouse liver, which was also verified in the insulin-resistant SMMC-7721 cells due to increased glucose uptake and activated AKT and AMPKα expression. Thus, the present study is the first to indicate that phycocyanin mediates antidiabetic function via activating the AKT and AMPK pathway in high glucose high fat diet induced T2DM mice and insulin-resistant SMMC-7721 cells, which lays a scientific theoretical basis for the potential treatment of diabetes and the utilization of marine natural products.

## 1. Introduction

Type 2 diabetes mellitus (T2DM), a chronic metabolic disorder characterized by decreased insulin secretion and insulin resistance, is becoming a major health issue worldwide [1]. T2DM is the most common form of diabetes, which accounts for 90% to 95% of all diabetic patients [2] and is expected to affect 439 million patients by 2030 [3].

Generally, T2DM results from the environmental interaction among genetic and other risk factors. Moreover, a loss of the first-phase of insulin release and increased glucagon secretion also accelerates the development of T2DM [4,5]. Multiple chemical drugs, including troglitazone, glyburide, miglitol, and metformin, are reported as therapeutic drugs for T2DM. However, their potential side effects cannot be ignored, such as the liver damage caused by long-term use [6]. Therefore, the search for safer and better antidiabetic agents has become an important area of active research.

Marine natural products with physiological activities have become novel functional materials for disease intervention. Phycocyanin, a blue photosynthetic pigment deriving from *Spirulina* and *Cyanobacteria* cells, has been used as a food additive in many products such as soft drinks, candies, chewing gum, and ice cream [7]. Meanwhile, phycocyanin is a type of functional factor that has been proven to possess therapeutic properties, including anti-inflammatory [8], antioxidant [9], antineoplastic [10], neuroprotective [11], and hepatoprotective [12] activities. Unlike most chemical drugs, phycocyanin is verified to have few or no toxic side effects [13,14]. So far, several studies have reported that phycocyanin could ameliorate glycometabolism levels in cell and animal models [15,16,17]. In particular, Ou et al. found the preventive effect of phycocyanin from *Spirulina platensis* on alloxan-injured mice [18], which revealed its beneficial effect in type 1 diabetes mellitus (T1DM). However, there is little information regarding the antidiabetic activity and regulatory mechanism of phycocyanin in T2DM [19,20,21].

In this work, we investigated the potential antidiabetic therapeutic functions of phycocyanin in a high glucose high fat diet induced diabetes mellitus mouse model as well as an insulin-resistant SMMC-7721 hepatoma carcinoma cell model. Moreover, the present work demonstrates that phycocyanin could attenuate T2DM via regulating the AKT and AMPK signaling pathway. This study lays the foundation for the application of phycocyanin in the field of T2DM and provides a scientific theoretical basis for the amelioration of T2DM.

## 2. Materials and Methods

### 2.1. Cell Line and Phycocyanin

An SMMC-7721 hepatoma carcinoma cell line [22] was preserved in the lab. Cells were cultured in DMEM (Invitrogen, Long Island, NY, USA) media supplemented with penicillin (100 units/mL), streptomycin (100 μg/mL), and 10% fetal bovine serum (FBS, HyClone, Logan, UT, USA). A phycocyanin sample was purchased from BinMei Biotechnology (Taizhou, China).

### 2.2. Establishment of Diabetes Mouse Model

All animal experiments were conducted strictly in accordance with the Guide for the Care and Use of Laboratory Animals approved by Pony Testing International Group (approval No. PONY-2021-FL-17). Male C57BL/6N mice (20–25 g, 4–6 weeks old) were obtained from Vital River Laboratory Animal Technology Co., Ltd. (Beijing, China) for one week of adaptive feeding (12 h light–dark cycle; 23 ± 2 °C; 45–60% relative humidity). The T2DM mouse model was established with a high-glucose high-fat diet combined with streptozocin (STZ) treatment. Briefly, after five weeks of high-glucose high-fat feeding and overnight fasting, mice were treated with 0.5% STZ (dissolved in citrate buffer) via intraperitoneal injection for four days, followed by high-glucose high-fat feeding for one week. The fasting blood glucose (FBG) content was analyzed using a blood glucose test kit. An FBG level above 11.1 mmol/L was considered to be diabetic and was used for further experiments. The negative control mice were treated with an equal dose of citrate buffer.

### 2.3. Experimental Design and Phycocyanin Intervention

The in vivo experimental design was performed as follows. The diabetic and control mice were randomly assigned to six groups with 12 mice per group:

NC group: normal mice received a normal diet only;
NCPC group: normal mice received normal diet and phycocyanin (200 mg/kg body weight);
T2DM model group: diabetic mice received a high-glucose high-fat diet;
TLPC intervention group: diabetic mice received a high-glucose high-fat diet and 100 mg/kg body weight phycocyanin;
THPC intervention group: diabetic mice received a high-glucose high-fat diet and 200 mg/kg body weight phycocyanin [18,23];
T group (positive control): diabetic mice received a high-glucose high-fat diet and 200 mg/kg body weight metformin.

The levels of fasting blood glucose, food intake, body weight, and sugar tolerance indexes were examined. After 10 weeks of intervention, the mice fasted overnight and were anaesthetized with 3% sodium pentobarbital. The liver and the pancreas tissues were excised and stored in liquid nitrogen. The serum was obtained by centrifugation at 1400× *g* for 10 min and stored at −80 °C.

### 2.4. Homeostasis Model Assessment-β (HOME-β) Detection

The HOME-*β* level reflects the function of pancreatic *β* cells. The HOME-*β* level was calculated based on the fasting serum insulin (FIN) and FBG levels according to the following formula:

HOME-*β* = 20 × (FIN)/(FBG − 3.5)

### 2.5. Biochemical Analysis

The fasting blood glucose level was detected using a blood glucose test kit (No. A154-1-1, Nanjing Jiancheng, Nanjing, China). The FIN level was detected using an insulin detection kit according to the indicated guidelines (No. JL11459, Jianglaibio, Shanghai, China). The levels of triglyceride (No. A110-1-1, TG), total cholesterol (No. A111-1-1, TC), aspartate transaminase (No. C010-2-1, AST), glutamic-pyruvic transaminase (No. C009-2-1, ALT), high-density lipoprotein (No. A112-1-1, HDL), low-density lipoprotein (No. A113-1-1, LDL), and superoxide dismutase (No. S0109, SOD) were detected using a biochemical detection kit (Beyotime, Shanghai, China). The histological observations of the liver and pancreas were carried out with HE staining.

### 2.6. Western Blot

SMMC-7721 cells and mouse liver tissues were collected and treated by RIPA. Cell lysates were separated by 12% SDS-PAGE at 120 V and were electrotransferred onto nitrocellulose membranes (Millipore, Burlington, MA, USA). The membranes were blocked with 5% nonfat dry milk in PBS, followed by incubation with the primary antibodies (1:1000) at 4 °C overnight and with horseradish peroxidase (HRP)-conjugated secondary antibodies (1:5000) for 30 min at 37 °C. *β*-actin was used as a control. The protein expression levels were analyzed using Image Lab™ Software on a ChemiDoc XRS+ (Bio-Rad, Hercules, CA, USA). The antibodies are purchased from Cell Signaling Technology (Boston, MA, USA). The product numbers of the antibodies were as follows: phospho-Akt pathway antibody (#9916), IRS antibody (#12879), phospho-AMPKα antibody (#50081), and phosphor-mTOR antibody (#5536).

### 2.7. Establishment of Insulin-Resistant SMMC-7721 Cell Model

SMMC-7721 cells were treated with different doses of insulin (10^−9^, 10^−8^, 10^−7^, 10^−6^, and 10^−5^ mol/L) for different treatment times (0, 12, 24, 36, 48, and 72 h). The level of the medium glucose content was examined by a glucose test kit. The glucose consumption of cells was calculated from the difference in the glucose content between the normal and treated groups.

### 2.8. Cell Viability Assay

SMMC-7721 cells were seeded at an appropriate density into 96-well plates. 3-(4,5-Dimethyl-2-thiazolyl)-2,5-diphenyl-2H-tetrazolium bromide (MTT) was used for viability detection. Briefly, MTT was added into each well for 4 h. Then, the DMSO was added for dissolution. The cell viability is presented as the ratio of the absorbance reading and the control cells. The absorbance was measured at 630 and 460 nm.

### 2.9. Statistical Analysis

The data were analyzed using Microsoft Excel. A two-tailed Student’s *t*-test was used to calculate the significant *p*-values. The results are presented as the means ± SD from three or more independent experiments. * or # *p* < 0.05 was considered significant; ** or ## *p* < 0.01 was considered extremely significant.

## 3. Results

### 3.1. Effects of Phycocyanin on Blood Glucose, Food Intake Levels, and Body Weight in Diabetic Mice

**Figure 1:**
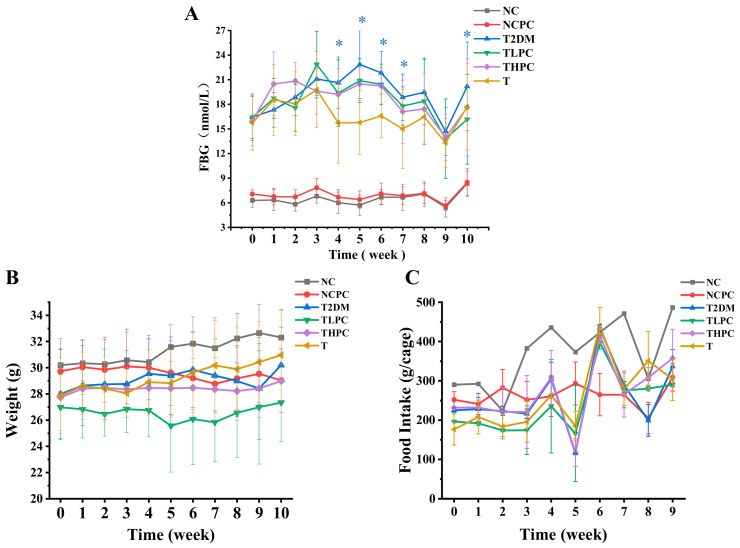
Effects of phycocyanin on blood glucose, body weight, and food intake levels in diabetic mice. (**A**) Detection of fasting blood glucose (nmol/L) of diabetic and phycocyanin-treated mice. (**B**) Detection of mouse body weight (g) under different treatments. (**C**) Detection of food intake (g/cage) of mice under different treatments. Data are expressed as means ± SD for 12 mice. * *p* < 0.05.

The FBG levels in mice were monitored each week (1–10 weeks). As shown in Figure 1A, the blood glucose in the NC and NCPC groups was maintained at the normal level throughout the determining period, indicating phycocyanin did not influence the FBG contents of mice. The FBG levels in phycocyanin-administrated diabetic mice (TLPC and THPC) were significantly decreased from the fourth week compared with the model group (T2DM), suggesting that the phycocyanin treatment could ameliorate the high glucose high fat diet induced diabetes of mice in a time-dependent manner. Although the hypoglycemic effect in the TLPC and THPC groups was not as obvious as metformin (T group), the FBG levels showed a gradually declining trend. In addition, the body weight and food intake analyses showed that phycocyanin and metformin treatments both had effects on the body weight and food intake levels in diabetic mice (Figure 1B,C), which was probably due to the inadaptability of the mice to external treatment. Taken together, these results indicate the protective function of phycocyanin against high glucose high fat diet induced diabetes mellitus in mice.

### 3.2. Effects of Phycocyanin on Glucose Tolerance, FIN, and HOME-β Levels in Diabetic Mice

**Figure 2:**
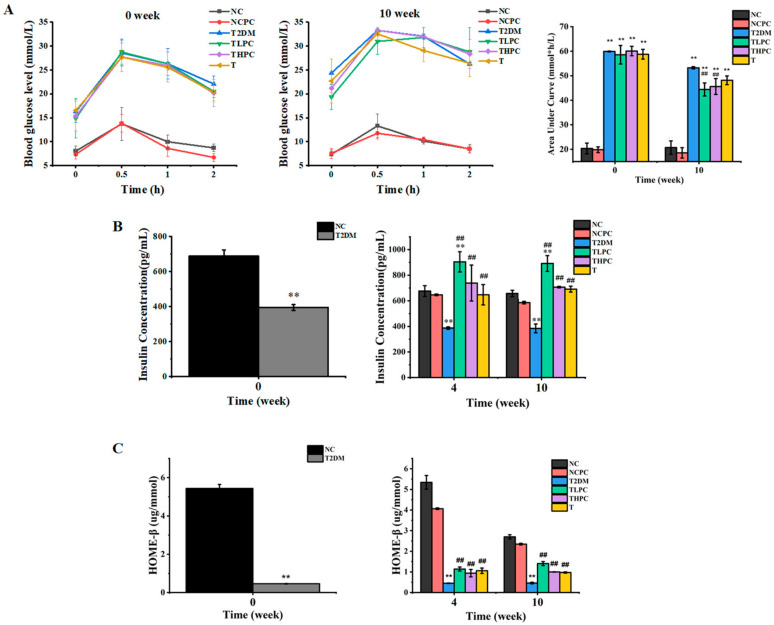
Effects of phycocyanin on glucose tolerance, FIN, and HOME-β levels in diabetic mice. (**A**) The glucose tolerance levels of diabetic mice under different treatments at week 0 and week 10. (**B**) The FIN levels of diabetic mice under different treatments at weeks 0, 4, and 10. (**C**) The HOME-β levels of diabetic mice under different treatments at weeks 0, 4, and 10. Data are expressed as means ± SD for 12 mice. ** (*p* < 0.01) represent groups with significant differences compared with the NC group. ## (*p* < 0.01) represent groups with significant differences compared with the T2DM group.

Blood glucose tolerance reflects the body’s ability to regulate the blood glucose concentration. In this study, the blood glucose content was examined within 2 h (0, 0.5, 1, and 2 h) after a fasting gavage of glucose in mice at week 0 and week 10. The areas under the curve are present in a histogram. As shown in Figure 2A, at week 0 the blood glucose tolerance levels in the T2DM, TLPC, THPC, and T groups were significantly higher than those in the NC and NCPC groups, and the four treatment groups showed similar glucose tolerances. At the 10th week, the blood glucose tolerance levels of the TLPC, THPC, and T groups were significantly lower than those in T2DM mice, indicating that phycocyanin could enhance the body’s tolerance to glucose. Figure 2B showed that the FIN level was obviously decreased in the T2DM mice compared with the NC group, revealing the impaired function of the pancreas. After phycocyanin intervention for 4 and 10 weeks, the FIN levels were significantly recovered compared to T2DM group. To further investigate whether phycocyanin exerted a protective function on pancreatic β cells, the HOME-β levels were analyzed. As shown in Figure 2C, the HOME-*β* levels were significantly decreased in the T2DM groups, suggesting the impaired function of pancreatic β cells in diabetic mice. However, phycocyanin (TLPC and THPC) could significantly recover the decreased HOME-β levels compared with the T2DM groups. These results indicate that phycocyanin exerts the function of maintaining the body’s glucose tolerance, recovering the decreased FIN levels and impaired pancreatic β cell function caused by a high-glucose high-fat diet in diabetic mice.

### 3.3. Phycocyanin Ameliorates the Biochemical Indicators of Serum in Diabetic Mice

**Figure 3:**
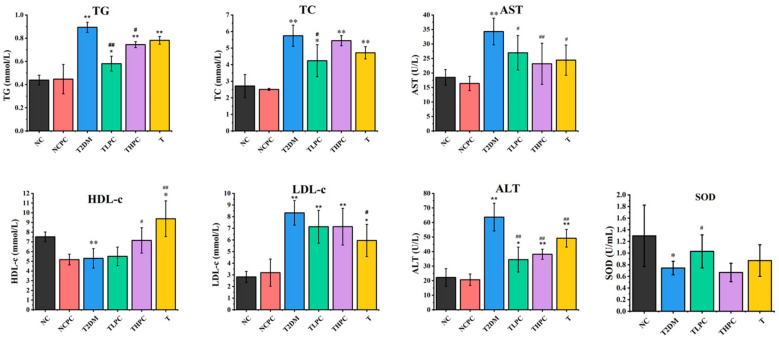
Phycocyanin ameliorates the biochemical indicators of serum in diabetic mice. The levels of triglyceride (TG), total cholesterol (TC), aspartate transaminase (AST), glutamic-pyruvic transaminase (ALT), high-density lipoprotein (HDL), low-density lipoprotein (LDL), and superoxide dismutase (SOD) were detected in the NC, NCPC, T2DM, TLPC, THPC, and T groups. Data are expressed as means ± SD for 12 mice. * (*p* < 0.05) and ** (*p* < 0.01) represent groups with significant differences compared with the NC group. # (*p* < 0.05) and ## (*p* < 0.01) represent group with significant differences compared with the T2DM group.

The blood biochemical index is a vital indicator to evaluate physical fitness. To investigate whether phycocyanin could recover the abnormal blood parameters in diabetic mice, multiple indicators were examined. Figure 3 shows that the levels of TG, TC, AST, LDL, and ALT in T2DM mice were extraordinary upregulated compared with the NC and NCPC groups, indicating that the aberrant blood biochemical index in diabetic mice was caused by liver metabolism failure. After phycocyanin administration, the contents of TG, TC, AST, and ALT in the TLPC and THPC groups were significantly decreased compared with the T2DM mice. Although there was no obvious difference in LDL between the phycocyanin treatment and T2DM groups, its content in the THPC and TLPC groups was slightly reduced, as shown in Figure 3. Meanwhile, the phycocyanin treatment promoted the levels of HDL, a type of healthy cholesterol in the blood, in TLPC mice compared with the T2DM group. In addition, phycocyanin (TLPC) could also reverse the decreased SOD expression in the blood of diabetic mice. Taken together, these results reveal that phycocyanin ameliorates the abnormal biochemical indicators of serum in diabetic mice.

### 3.4. Phycocyanin Restores the Organizational Integrity of the Liver and Pancreas in Diabetic Mice

**Figure 4:**
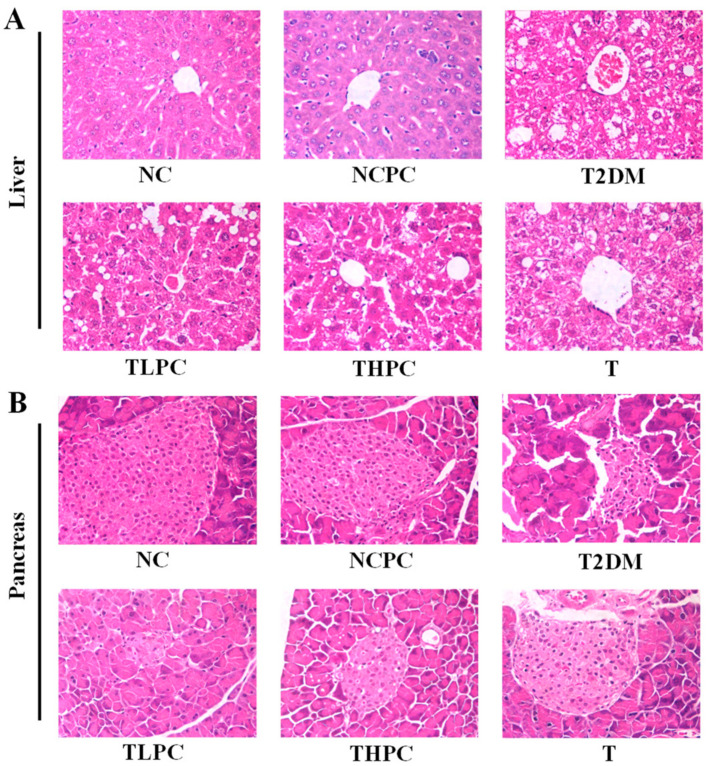
Phycocyanin restores the organizational integrity of the liver and pancreas in diabetic mice. (**A**) Morphological features of mouse liver in different treatment groups. (**B**) Morphological features of mouse pancreas in different treatment groups.

The tissue morphological results are shown in Figure 4. Liver tissues in the NC and NCPC groups showed typical hepatic lobule structures, including sinusoids, plates of hepatocytes, and central veins (Figure 4A). By contrast, the liver tissue in the T2DM group showed impaired hepatocytes and blocked veins. After phycocyanin administration, the impaired hepatic lobule structure was restored compared with the T2DM group. Likewise, the control pancreatic tissues showed septal and intralobular duct distribution as well as conjunctive tissue lobules (Figure 4B). In the T2DM group, pancreatic tissues showed depauperate pancreatic islets, few traces of endocrine tissue, and an obscure demarcation between islets and the surrounding acini. However, phycocyanin could improve the morphology of pancreatic islets and restored the number of pancreatic cells. These results indicate that phycocyanin could restore the organizational integrity of the liver and pancreas in diabetic mice.

### 3.5. Phycocyanin Promotes the Activity of the AKT and AMPK Pathway in Diabetic Mice

**Figure 5:**
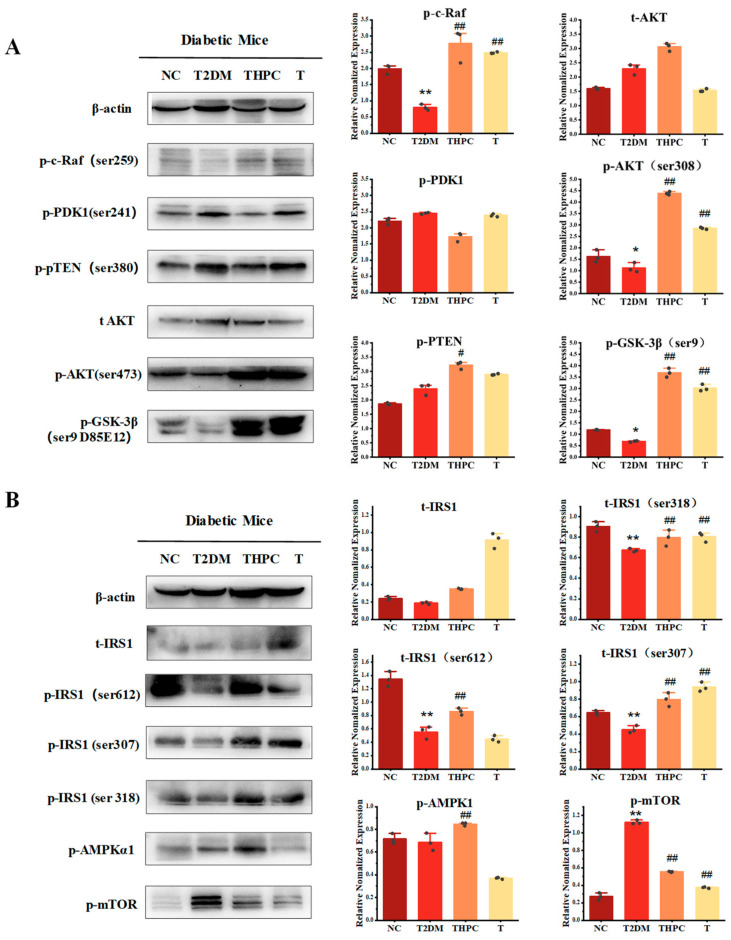
Phycocyanin promotes the activity of the AKT and AMPK pathway in diabetic mice. (**A**) Western blot analysis of the AKT signaling pathway in the NC, T2DM, THPC, and T groups. (**B**) Western blot analysis of the expressions of the AMPKα and IRS proteins in the NC, T2DM, THPC, and T groups. Data are expressed as means ± SD for random three mice. * (*p* < 0.05) and ** (*p* < 0.01) represent groups with significant differences compared with the NC group. # (*p* < 0.05) and ## (*p* < 0.01) represent groups with significant differences compared with the T2DM group. The dots in the histogram represent the three different grey values of the Western blot samples.

To further explore the underlying regulatory mechanism of phycocyanin in diabetic mice, the activities of the corresponding pathways involved in cellular metabolism were examined. The expressions of AKT signaling pathway proteins in mouse liver are shown in Figure 5A. PTEN is an AKT inhibitor protein located upstream of AKT signaling, which is activated through phosphorylation [24], while GSK-3β is a downstream protein of the AKT pathway, which is regulated by AKT [25]. In the T2DM diabetic mouse group, the phosphorylated PTEN was upregulated, while the phosphorylation levels of c-Raf, AKT, and GSK-3β were significantly decreased compared with the control group, revealing that the AKT pathway activity in the diabetic mouse liver was attenuated. By contrast, the phycocyanin treatment obviously reversed the phosphorylation levels of these proteins, as shown in the THPC and T groups. The above results indicate that phycocyanin is likely to activate AKT signaling in diabetic mice, promote the availability of glucose, and alleviate the diabetic symptoms of mice.

Besides the AKT pathway, the expressions of the AMPKα, mTOR, and IRS proteins were also analyzed in this work. IRS1 is the receptor substrate of insulin, which plays important roles in glucose utilization [26]. As shown in Figure 5B, the phosphorylation levels of IRS1 (ser612, ser307, and ser318) were decreased in T2DM mice, while phycocyanin treatment could restore their expressions. In addition, phosphorylated AMPKα expression was significantly upregulated, while phosphorylated mTOR was suppressed in the THPC group compared with the T2DM group. It was reported that AMPK is activated by a falling cellular energy status but suppressed upon overnutrition [27]. In this case, phycocyanin is most likely to enhance the sensibility of AMPK on blood glucose and promote the utilization of glucose in mice.

### 3.6. Phycocyanin Accelerates the Glucose Uptake of Normal and Insulin-Resistant SMMC-7721 Cells

**Figure 6:**
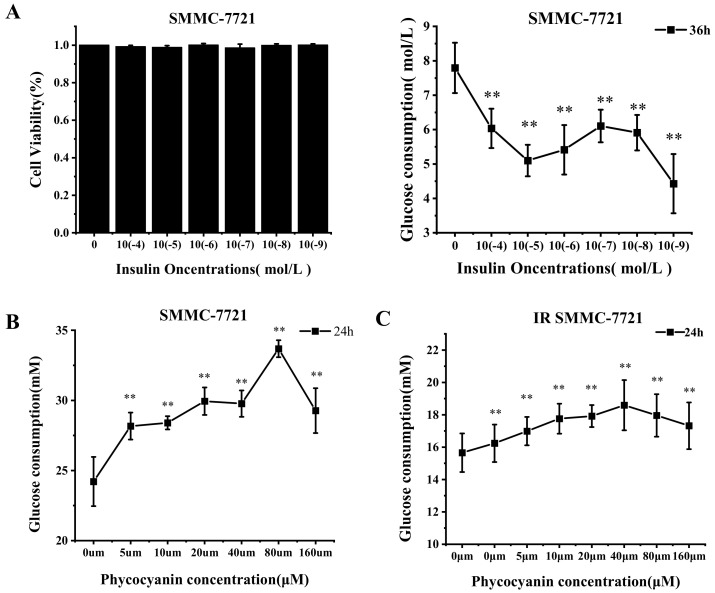
Phycocyanin accelerates the glucose uptake of insulin−resistant SMMC-7721 cells. (**A**) Cell viability and glucose consumption assays of SMMC-7721 cells after different doses of insulin treatment. (**B**) Glucose consumption assay of SMMC-7721 cells after different doses of phycocyanin treatment. (**C**) Glucose consumption assay of insulin-resistant SMMC-7721 cells after different doses of phycocyanin treatment. Data are expressed as means ± SD. ** (*p* < 0.01) represent groups with significant differences compared with the NC group.

SMMC-7721 cells were selected to further verify the results of the mouse model. First, the insulin-resistant SMMC-7721 cell line was constructed via exposure to insulin. As shown in Figure 6A, insulin treatment (0, 10^−4^, 10^−5^, 10^−6^, 10^−7^, 10^−8^, and 10^−9^) did not affect the viability, while it significantly inhibited the glucose consumption of SMMC-7721 cells, indicating that insulin-resistant SMMC-7721 cell lines were constructed. According to Figure 6A, an insulin treatment dose of 10^−5^ was selected for the following study. To investigate whether phycocyanin could reverse the insulin-induced decreased glucose uptake of cells, the glucose consumption was measured after phycocyanin treatment in normal and insulin-resistant SMMC-7721 cells. As shown in Figure 6B, phycocyanin exposure significantly promoted the glucose consumption of normal SMMC-7721 cells in a dose-dependent manner. Strikingly, in insulin-resistant SMMC-7721 cells, the glucose uptake was also increased due to the treatment of phycocyanin. Taken together, these results reveal that phycocyanin accelerates the glucose uptake of both normal and insulin-resistant SMMC-7721 cells, which supports the results of the mouse model.

### 3.7. Phycocyanin Increases the Activity of the AKT and AMPK Pathway in Insulin-Resistant SMMC-7721 Cells

**Figure 7:**
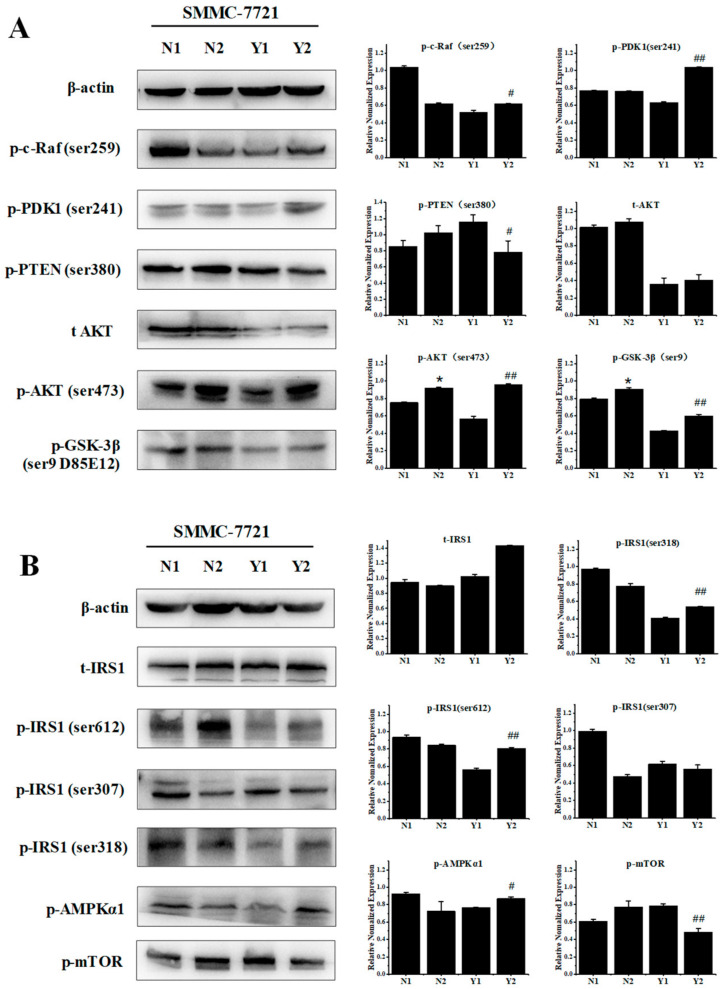
Phycocyanin increases the activity of the AKT and AMPK pathway in insulin-resistant SMMC-7721 cells. (**A**) Western blot analysis of the AKT signaling pathway in the N1, N2, Y1, and Y2 groups. (**B**) Western blot analysis of the expressions of the AMPKα and IRS proteins in the N1, N2, Y1, and Y2 groups. * (*p* < 0.05) represent groups with significant differences compared with the N1 group. # (*p* < 0.05) and ## (*p* < 0.01) represent groups with significant differences compared with the Y1 group. N1 represents control cells, N2 represents control cells with phycocyanin treatment, Y1 represents insulin-resistant cells, and Y2 represents insulin-resistant cells with phycocyanin treatment.

The AKT and AMPK pathway activities were also analyzed in the insulin-resistant SMMC-7721 cell model. As shown in Figure 7A, the AKT signaling levels were analyzed in normal (N1), phycocyanin-treated normal (N2), insulin-resistant (Y1), and phycocyanin-treated insulin-resistant (Y2) SMMC-7721 cells. In accord with the mouse results, the phosphorylation levels of c-Raf, PDK1, AKT, and GSK-3β were significantly upregulated in the Y2 group compared with the Y1 group. Meanwhile, the phosphorylated PTEN levels were decreased after phycocyanin treatment in the Y2 group. In addition to the AKT pathway, the phosphorylation levels of IRS1 (ser612 and ser307) were promoted in the Y2 group compared with the Y1 group (Figure 7B). In addition, phosphorylated AMPKα expression was significantly upregulated, while phosphorylated mTOR was suppressed in the Y2 group compared with the Y1 group. These results indicate that phycocyanin could increase the activity of the AKT and AMPK pathway in insulin-resistant SMMC-7721 cells, which further confirms the mouse model results.

## 4. Discussion

Many studies have reported that phytochemicals can ameliorate obesity-induced diabetes mellitus in vivo and in vitro [28,29]. In this study, we have demonstrated that phycocyanin can protect against high glucose high fat diet induced diabetes mellitus in mice and can also ameliorate the insulin-resistant effect of SMMC-7721 cells. Furthermore, phycocyanin can significantly reverse the decreased AKT signaling of diabetes mellitus mice and insulin-resistant SMMC-7721 cells. In addition, the AMPK activity was also promoted after phycocyanin exposure in vivo and in vitro.

STZ is considered the most potent diabetogenic chemical used in research and can be used for establishing both T1DM and T2DM models. It is reported that the administration of multiple low doses of STZ to mice for several days is more likely to induce a T1DM model due to the partial damage to pancreatic islets and the inflammatory process [30]. However, a high-fat diet combined with STZ administration is a good way to establish a T2DM model, which was employed in our work. Besides STZ, alloxan is another potent diabetogenic chemical in most studies. In this study, we employed a high-glucose high-fat diet combined with STZ treatment to establish the diabetic mouse model. Zucker diabetic fatty (ZDF) and Goto-Kakizaki rats are better T2DM animal models in studies, which could exhibit symptoms including diabetes, insulin resistance, hyperlipidemia, and various complications [31]. Nevertheless, the present results indicate a preventive and protective function of phycocyanin in the T2DM mice.

Phycocyanin, ubiquitous in cyanobacteria, is widely utilized in food, cosmetics, and biochemical pharmaceuticals. It is also a potent antioxidant with great biological activities [32]. Although phycocyanin has been reported to be effective in diabetic animals, little has been demonstrated in terms of its function in high glucose high fat diet induced mice and insulin-induced SMMC-7721 cells. It was reported that *Spirulina maxima* extract could reduce obesity through the suppression of adipogenesis and the activity of browning in 3T3-L1 cells and high fat diet induced obese mice [15], which was partially in line with our present results. It is worth noting that phycocyanin could recover the alloxan-induced decreased fasting serum insulin levels in mice [23]. In this case, our present study has completed the theory that phycocyanin improves diabetes mellitus, including T1DM and T2DM.

AMPKα plays a pivotal role in metabolic regulation such as glycometabolism, autophagy, cell movement, and protein synthesis [33]. It has been proven that the activation of AMPKα can decrease the body weights of HFD-induced obesity mice [34], which indicates the metabolism-promoting function of AMPKα. Generally, AMPK signaling is activated upon the state of malnutrition and hunger then promotes protein hydrolysis and glycolysis and maintains the blood glucose balance [35]. Thus, the prometabolic activity of AMPK is one of the important regulatory mechanisms for cell survival. In particular, the present work displayed that the phosphorylation levels of AMPKα were increased in both the diabetes mellitus mouse and insulin-resistant SMMC-7721 cell models, indicating that phycocyanin could enhance the sensibility of insulin and promote the uptake and utilization of glucose by activating AMPKα. Meanwhile, activated AMPK promotes autophagy directly by phosphorylating multiple autophagy-related proteins [36]. Coincidentally, our previous study demonstrated that phycocyanin diminished the viability of non-small-cell lung cancer cells via the induction of autophagy by downregulating p-mTOR expression [37], which was in line with the present results.

AKT is a serine/threonine kinase, which participates in the key role of the PI3K signaling pathway. AKT can be activated by multiple growth signals and then modulates the function of downstream proteins involved in proliferation, cellular survival, metabolism, migration, and angiogenesis [38]. It has been reported that hyperglycemia, dyslipidemia, inflammation, and insulin resistance in T2DM were ameliorated after the oral administration of Scutellariae Radix and Coptidis Rhizoma, two types of traditional Chinese medicines, via regulating the PI3K/AKT signaling pathway [39], indicating that AKT activation was involved in glycometabolism regulation. Similar results were verified in a T2DM KKAy mouse model [40]. In particular, Gao et al. investigated the protective effects of phycocyanin on insulinoma β cells against MG-induced cell dysfunction and discovered that phycocyanin could protect INS-1 pancreatic β cells by modulating the PI3K/AKT pathway [41]. In this work, AKT was significantly activated after phycocyanin treatment in T2DM mice and insulin-resistant SMMC-7721 cell models, which provided the underlying mechanism of phycocyanin in the process of ameliorating diabetes.

In summary, our study shows that phycocyanin ameliorates high glucose high fat diet induced diabetes mellitus mice by downregulating fasting blood glucose and serum biochemical indicators, including TG, TC, AST, and ALT, as well as promoting the glucose tolerance and fasting serum insulin levels of diabetic mice. Meanwhile, phycocyanin can restore the organizational integrity of the liver and pancreas in diabetic mice. In addition, a molecular analysis revealed that phycocyanin protects against diabetes mellitus by activating the AKT and AMPK signaling pathway in both the high glucose high fat diet induced mouse and insulin-resistant SMMC-7721 cell models. To the best of our knowledge, this work is the first to show the roles phycocyanin in T2DM and to illustrate the underlying mechanisms, which involved activating the AKT and AMPK pathway in vivo and in vitro. As phycocyanin is a type of natural functional food with few side effects on cells, it could be used as a dietary nutrient material with a blood-glucose-regulating function. The results would lay a scientific theoretical basis for the treatment of diabetes and the utilization of marine natural products.

## Data Availability

Data presented in this article are available at request from the corresponding author.
